# Thyroid collision tumor containing oncocytic carcinoma, classical and hobnail variants of papillary carcinoma and areas of poorly differentiated carcinoma

**DOI:** 10.20945/2359-3997000000389

**Published:** 2021-07-16

**Authors:** Marcos Tadashi Kakitani Toyoshima, Regina Barros Domingues, Ibere Cauduro Soares, Debora Lucia Seguro Danilovic, Larissa Costa Amorim, Edla R. C. Cavalcante, Fernanda F. Antonacio, Felipe Santa Rosa Roitberg, Ana Oliveira Hoff

**Affiliations:** 1 Universidade de São Paulo Faculdade de Medicina Hospital das Clínicas São Paulo SP Brasil Serviço de Onco-Endocrinologia, Instituto do Câncer do Estado de São Paulo Octavio Frias de Oliveira, Hospital das Clínicas, Faculdade de Medicina, Universidade de São Paulo, São Paulo, SP, Brasil; 2 Universidade de São Paulo Faculdade de Medicina Hospital das Clínicas São Paulo SP Brasil Departamento de Patologia, Instituto do Câncer do Estado de São Paulo Octavio Frias de Oliveira, Hospital das Clínicas, Faculdade de Medicina, Universidade de São Paulo, São Paulo, SP, Brasil; 3 Universidade de São Paulo Faculdade de Medicina Hospital das Clínicas São Paulo SP Brasil Departamento de Oncologia Clínica, Instituto do Câncer do Estado de São Paulo Octavio Frias de Oliveira, Hospital das Clínicas, Faculdade de Medicina, Universidade de São Paulo, São Paulo, SP, Brasil

## Abstract

Collision tumors are rare and may comprise components with different behavior, treatments, and prognosis. We report an unprecedented case of aggressive thyroid collision tumor containing widely invasive oncocytic carcinoma (OC), classical and hobnail (HPTC) variants of papillary carcinoma, and poorly differentiated carcinoma (PDTC). The patient underwent total thyroidectomy, radioactive iodine therapy, and within months progressed with local recurrence, and pulmonary metastases requiring neck dissection, external radiotherapy and systemic treatment with sorafenib. The rapid progression, dedifferentiated metastatic lesions, and failure to treatments resulted in the patient´s death. The great variety of histological types and the evolution of this case were a challenge for the management of metastatic disease. Widely invasive OC, HPTC and PDTC are considered to have a worse prognosis. HPTC has never been reported as a component of a collision tumor. HPTC and PDTC should call attention to a possible higher-grade transformation.

## INTRODUCTION

Differentiated thyroid carcinomas (DTC) are the most prevalent malignant endocrine tumors ( 1 ). Papillary (PTC) and follicular (FTC) thyroid carcinomas are the most prevalent DTC. In general, thyroid cancer has a good prognosis with 10-year overall survival rates for patients with PTC and FTC of 93% and 85%, respectively ( 2 ). However, some histopathological variants of follicular cell-derived thyroid cancer, such as (i) tall cell, (ii) columnar cell and (iii) hobnail variants of PTC; (iv) widely invasive FTC; and (v) poorly-differentiated carcinoma (PDTC) are associated with more unfavorable outcomes, including increased risk of tumor-related death ( 3 ).

Oncocytes, also known as Hürthle cells, are large, polygonal, epithelial cells with an acidophilic cytoplasm, containing a great number of mitochondria, large hyperchromatic nucleus, and prominent nucleolus. Oncocytes are associated with benign conditions, as lymphocytic thyroiditis, Graves disease, and hyperplastic nodules in multinodular goiters. Malignant diseases that are associated with oncocytes include an oncocytic variant of PTC and minimally invasive or widely invasive oncocytic carcinoma (OC) ( 4 , 5 ). OC accounts for about 5% of differentiated thyroid cancers ( 6 , 7 ) and was previously considered to be a variant of FTC; however, after thorough genomic analysis, it has been considered distinct from FTC ( 8 ). In addition, OC has a more aggressive behavior and is less avid to radioactive iodine compared to FTC ( 6 ). The metastatic spread of OC is mainly hematogenous, and therefore lymph node metastases are less common than PTC. The risk of recurrence of widely invasive OC is 73% ( 5 ) and the estimated 5- and 10-year disease-specific survival rates of metastatic OC are 81% and 60%, respectively ( 7 ).

In 2004, a Japanese study ( 9 ) showed that the loss of the cell polarity (hobnail appearance) could be a characteristic of poor cell differentiation and an increased risk of tumor recurrence in PTC. However, the hobnail variant of PTC (HPTC) was only described in 2009 ( 10 ), and since then, more than 100 HPTC cases have been described ( 11 – 13 ). Currently, HPTC is a recognized aggressive variant of PTC. The loss of polarity/cohesiveness with hobnail features in ≥30% of tumor cells is one of the criteria for the diagnosis of HPTC ( 11 ). PTC with hobnail/micropapillary characteristics (<30% of tumor cells with hobnail features) appears to be more aggressive than classic papillary carcinoma ( 14 ).

PDTC is an aggressive thyroid tumor, characterized by a partial loss of thyroid differentiation. Its morphological and behavioral characteristics are intermediate between DTC and anaplastic carcinoma. PDTC causes locally invasive cervical disease in more than 50%, metastasis to lymph nodes (LNs) in 50% to 85%, and distant metastasis in up to 85% of cases. Five-year disease-specific survival for PDTC patients has been reported at 66% ( 15 ).

Collision tumors, defined as two or more neoplasms coexisting in one anatomical site with distinct histology, are rare ( 16 ). Thyroid collision tumors are rare, and just over 40 case reports have been described ( 16 – 23 ).

We report an unprecedented case of aggressive thyroid collision tumor containing OC, classical and hobnail variants of PTC, and areas with PDTC.

## CASE REPORT

**Figure 1:**
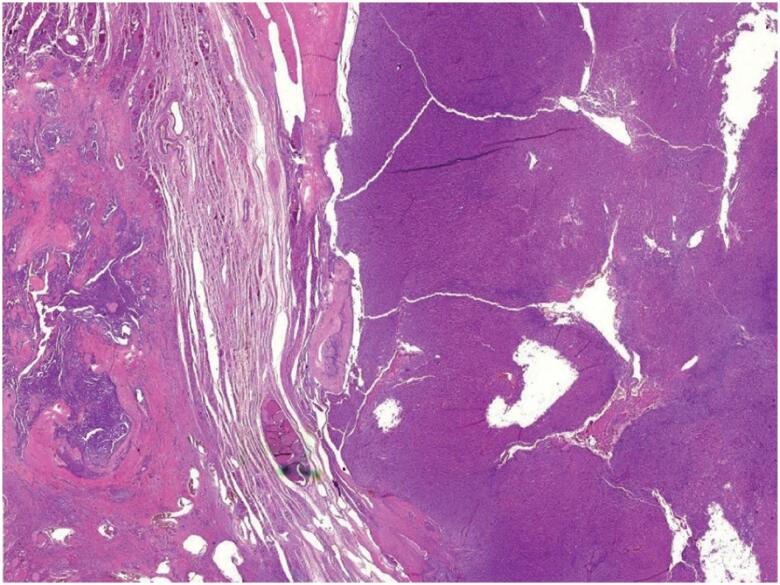
Photomicrograph of the surgical specimen showing stain, showing two components of the collision tumor. On the right side of the figure, oncocytic carcinoma with capsular invasion and on the left side, classic variant of papillary thyroid carcinoma (H&E, 25X).

**Figure 2:**
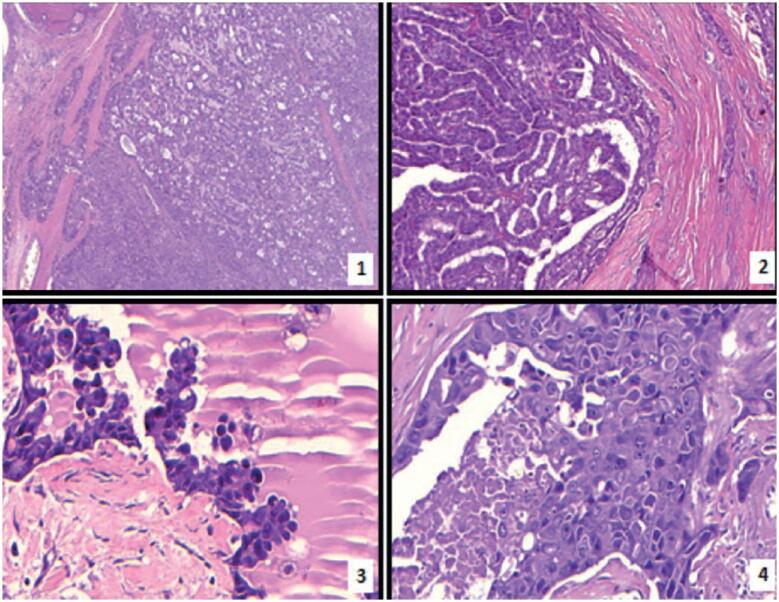
Photomicrographs of the tumor. Histopathological features of the four components of the collision tumor: ( **1** ) Oncocytic carcinoma with capsular invasion (H&E, 40x); ( **2** ) Conventional (classic) papillary carcinoma (H&E, 100x); ( **3** ) Hobnail variant of papillary thyroid carcinoma (H&E, 400x); ( **4** ) Poorly differentiated thyroid carcinoma (H&E, 400x).

**Figure 3:**
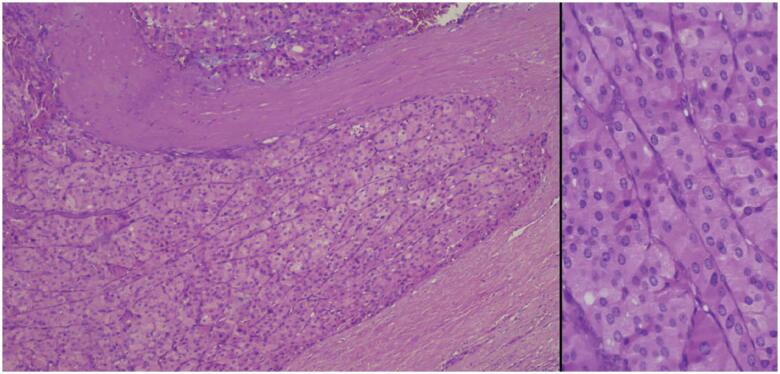
Higher magnification of oncocytic carcinoma with capsular invasion (H&E, 100x, 400x at right).

**Figure 4:**
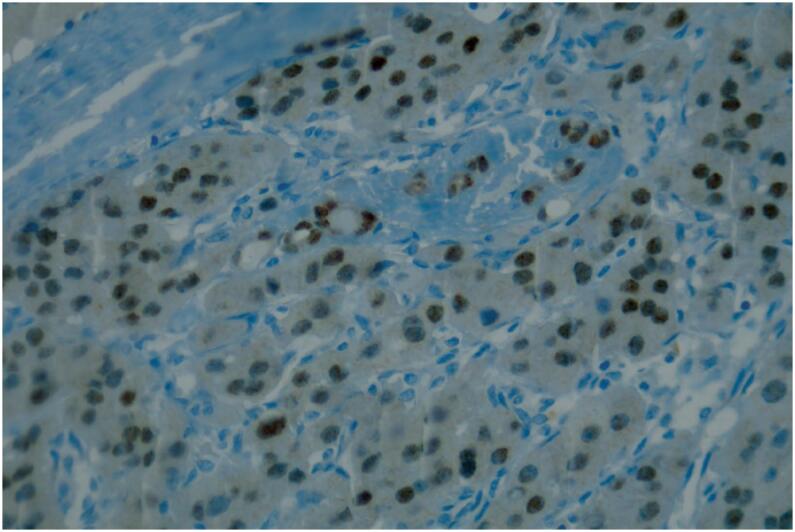
PAX8 positivity at oncocytic carcinoma (immunohistochemistry, 400x).

A 63-year-old woman with a long-standing history of thyroid nodules was referred to our hospital. She knew the diagnosis of thyroid nodules for 25 years. Thyroid ultrasonography (US) showed heterogeneous nodules with gross calcifications in the left lobe of the thyroid, the largest being 4.9 × 6.4 × 3.5 cm. A fine-needle aspiration (FNA) biopsy of this nodule revealed a follicular neoplasm with oncocytes (Bethesda category IV). The patient refused surgical treatment for 4 years, when she developed pain and dysphagia. She was then submitted to total thyroidectomy, and the histopathologic report depicted three components in the left lobe of the thyroid: widely invasive OC, measuring approximately 6.0 cm in the longest axis, in addition, classic variant PTC (CVPTC), but with hobnail component and foci of poorly-differentiated carcinoma ( Figure 1 , Figure 2 and Figure 3 ). The CVPTC measured 0.8 cm and the set of hobnail and poorly differentiated components measured 1.8 cm in the longest axis. The hobnail component of papillary carcinoma was characterized by the presence of papillary and micropapillary structures lined by cells with decreased nucleus-cytoplasm ratio, loss of cohesiveness, eosinophilic cytoplasm, apical nuclei and with evident nucleoli. The poorly differentiated component was characterized by the presence of solid blocks of cells, but without nuclear characteristics of papillary carcinoma, exhibiting intense atypia, vesicular nuclei with evident nucleoli, presence of mitoses (3 mitoses in 10 high magnification fields) and necrosis. Surgical margins were free of disease, and there was no extrathyroidal extension, angiolymphatic invasion, nor perineural invasion. Adenomatous goiter with areas of follicular hyperplasia, associated with chronic lymphocytic thyroiditis, was found around these tumors. The 7^th^ American Joint Committee and International Cancer Control and the American Joint Committee on Cancer (AJCC/UICC) TNM (tumor-node-metastasis) thyroid cancer staging was defined as pT3pNxpMx. The immunohistochemical profile (IHC) revealed focal thyroglobulin staining in all tumoral components (OC, CVPTC, HPTC and PDTC); TTF-1 positive in OC, and inconclusive in PTC; PAX8 was focal positive in OC ( Figure 4 ), and inconclusive in CVPTC; p53 positive staining was observed in OC, HPTC and PDTC, and inconclusive in CVPTC.

*BRAF* mutational analysis by gene sequencing was performed on samples of all components of thyroid carcinoma, but no mutations were found.

The patient underwent radioactive iodine therapy (RAI) with ^131^I with 206 mCi (7622Mbq) upon thyroid hormone withdrawal. Serum TSH and thyroglobulin (TG) before RAI were 91.08 mcU/mL and 6.0 ng/mL, respectively. The post-treatment ^131^I whole-body scan showed uptake in thyroid bed and possible central compartment neck LNs. The radioactive iodine uptake was 1.5%.

Six months later at a follow-up visit, serum TG and anti-TG antibodies were undetectable, neck US revealed heterogeneous LNs ranging from 1.3 to 1.6 cm in level IV bilaterally which were biopsied; cytology from both LNs was consistent with metastatic carcinoma, with morphological aspect similar to the PDTC, despite an undetectable TG from both LNs aspirate. The IHC panel showed positive focal immunoexpression for TTF-1, and negative for thyroglobulin and PAX8.

In the face of a recurrence from a poorly-differentiated tumor, a positron emission tomography-computed tomography (PET-CT) with fluorine-18 fluorodeoxyglucose (^18^F-FDG) was obtained showing not only both LNs identified by the US (SUVmax 16.5%) but also several pulmonary nodules, some cavitating, the largest with 2.2 cm in its largest diameter (SUVmax 5.6%).

Lung biopsy revealed the presence of papillary pattern adenocarcinoma, with positive immunoexpression only for cytokeratin 7 (CK7), inconclusive for PAX8 and P53 and negative for thyroglobulin and TTF-1. Other markers indicative of origin in the breast, lower digestive tract or lungs were negative. The immunoexpression of PAX8 and p53 proved to be inconclusive.

Because of the significant progression of local disease, she underwent bilateral LN dissection (levels II-V), which path report revealed metastasis in 6 out of 49 LNs with extensive extracapsular invasion involving fibroadipose tissue and skeletal muscle and a 5-cm soft tissue metastasis infiltrating skeletal muscle from levels II-IV on the left. IHC was consistent with a poorly-differentiated carcinoma of unknown primary source (p16, p63, CK7 and GATA-3 positive, c-erb-B2/HER-2, p53, WT1, BRST2, Cdx2, CK20 cytokeratin, PAX8, estrogen receptor, thyroglobulin, TTF-1 all negative). Within a month from this surgical procedure, she developed acute respiratory failure requiring emergency tracheostomy. Staging workup (computed tomography scan of the head, neck, and chest and ^18^F-FDG PET-CT) showed evidence of local recurrence, enlargement of pulmonary nodules, and new liver metastases. She underwent external-beam radiotherapy to the cervical area (cumulative dose of 20Gy) and initiated treatment with sorafenib 400mg twice a day with good tolerance but with no therapeutic response. She died two months later with the rapid progression of the pulmonary metastases.

## DISCUSSION

We report a case of a patient with a collision tumor containing widely invasive OC, classical and hobnail variants of PTC, and areas with PDTC. Collision tumors can contain components with different aggressiveness, treatments, and prognosis, challenging their management ( 24 ). Widely invasive OC, HPTC, and PDTC are some of the thyroid cancers that are considered to have a worse prognosis among DTC ( 3 ).

Several hypotheses have been suggested as mechanisms for collision tumors: (i) a simple coincidence, (ii) one tumor predisposing to the other, (iii) a carcinogenic factor predisposing the tumors involved, or (iv) tumors that derive from stem cell remnants ( 17 ). Most reports of thyroid collision tumors consist of papillary and medullary thyroid carcinomas ( 16 ).

Our literature review found five case reports of collision tumors containing PTC and OC ( 19 , 25 – 27 ). As in our case report, three works also reported association with Hashimoto's thyroiditis ( 19 , 26 , 27 ). However, the relationship between Hashimoto's thyroiditis and the pathogenesis of thyroid cancer, especially PTC, remains controversial ( 28 ).

HPTC is a rare and quite aggressive variant of PTC and has not yet been described as a component of a collision tumor of the thyroid. The hobnail features have been reported in cases of poorly-differentiated and anaplastic thyroid carcinoma. The presence of these features should alert to a possible higher-grade transformation ( 29 ).

PDTC is associated with an increased risk of metastasis and tumor-related death ( 3 , 15 ). The presence of PDTC in the collision tumor is a sign of aggressiveness, poor prognosis and likely transformation of the more differentiated PTC ( 24 ).

The somatic mutation burden is relatively low in thyroid tumors, making it easier to understand their pathogenesis. Driver mutations are identified in more than 90% of thyroid cancers, and passenger mutations that can modify the biological behavior of the tumor can occur in many tumors ( 30 ). The main genes with mutations in the HPTC are *BRAF* and *TP53. RET/PTC1* rearrangements and in the *TERT* promoter mutations have also been reported ( 13 ). The most common mutations in PDTC are *TERT, BRAF, RAS* , and *TP53* mutations ( 15 ). The sample size of the cancer subtypes was limited for the genetic analysis of our patient, permitting only the analysis of the *BRAF* gene, which resulted without mutations. Genetic and epigenetic changes are involved in the initiation, progression, and dedifferentiation of cancers, including thyroid cancer. *BRAF* and *RAS* mutations are more involved in an earlier event of the tumorigenesis, and mutations in the *TP53* and *CTNNB1* genes, for example, in later events ( 1 ). This case called our attention to the rapid and severe progression of the disease. It led us to raise the hypothesis of the dedifferentiation of the metastases ( 1 ) and the successive transformations of the tumor, possibly from classical variant PTC to HPTC and from HPTC to PDTC. Unfortunately, a more thorough genetic analysis of the tumor was not possible. However, the IHC profile showing positivity for p53 in HPTC, and in poorly-differentiated carcinoma components suggest gaining of poor prognostic mutations. The lack of response to therapy and the short survival time observed in this case was similar to the expected survival observed in anaplastic thyroid carcinoma. However, it was not detected in any of the performed biopsies.

To conclude, this is a very rare case report of a thyroid collision tumor involving several histological patterns of carcinoma, including PTC with hobnail characteristics and possible progression of the component of PDTC with a very aggressive evolution.
